# In Vitro Assessment of Organic and Residual Fractions of Nematicidal Culture Filtrates from Thirteen Tropical *Trichoderma* Strains and Metabolic Profiles of Most-Active

**DOI:** 10.3390/jof8010082

**Published:** 2022-01-15

**Authors:** Felicia Amalia Moo-Koh, Jairo Cristóbal-Alejo, María Fé Andrés, Jesús Martín, Fernando Reyes, Jose María Tun-Suárez, Marcela Gamboa-Angulo

**Affiliations:** 1 Centro de Investigación Científica de Yucatán, A. C. Calle 43 No. 130, Col. Chuburná de Hidalgo, Mérida 97205, Mexico; famk22@hotmail.com; 2 Tecnológico Nacional de México, Campus Conkal, Avenida Tecnológico s/n, Conkal 97345, Mexico; jose.tun@itconkal.edu.mx; 3 Instituto de Ciencias Agrarias, CSIC, Serrano 115-dpdo, 28006 Madrid, Spain; mafay@ica.csic.es; 4 Fundación MEDINA, 18016 Granada, Spain; jesus.martin@medinaandalucia.es (J.M.); fernando.reyes@medinaandalucia.es (F.R.)

**Keywords:** ethyl acetate fraction, HRMS dereplication, *Trichoderma ghanense*, *Trichoderma harzianum*, *Trichoderma koningiopsis*, *Trichoderma virens*

## Abstract

The nematicidal properties of *Trichoderma* species have potential for developing safer biocontrol agents. In the present study, 13 native *Trichoderma* strains from *T. citrinoviride*, *T. ghanense* (2 strains), *T. harzianum* (4), *T*. *koningiopsis*, *T. simmonsii*, and *T. virens* (4) with nematicidal activity were selected and cultured in potato dextrose broth to obtain a culture filtrate (CF) for each. Each CF was partitioned with ethyl acetate to obtain organic (EA) and residual filtrate (RF) fractions, which were then tested on second-stage juveniles (J2s) of the nematodes *Meloidogyne javanica* and *M. incognita* in a microdilution assay. The most lethal strains were *T. harzianum* Th43-14, *T. koningiopsis* Th41-11, *T. ghanense* Th02-04, and *T. virens* Th32-09, which caused 51–100% mortality (%M) of J2s of both nematodes, mainly due to their RF fractions. Liquid chromatography–diode array detector-electrospray-high resolution mass spectrometry analysis of the most-active fractions revealed sesquiterpene and polyketide-like metabolites produced by the four active strains. These native *Trichoderma* strains have a high potential to develop safer natural products for the biocontrol of *Meloidogyne* species.

## 1. Introduction

Fungi belonging to the *Trichoderma* genus are cosmopolitan species, with 488 species identified [1]. Several of these species have been widely studied as biocontrol agents against phytopathogenic fungi [2] and nematodes [3,4], insects [5], and weeds [6], and as plant growth promoters [7,8]. The nematicidal potential of *Trichoderma* species is increasingly being harnessed to develop new and safer biocontrol agents against parasitic nematodes such as *Globodera pallida*, *Heterodera avenae*, *Meloidogyne incognita*, *M*. *javanica*, *M. hapla*, and *Pratylenchus brachyurus* [8,9]. In particular, *Meloidogyne* root-knot nematodes are considered the most harmful because they can affect a wide range of crops, causing production losses between 25% to 50% and millions of dollars. They thus continue to be controlled mainly with synthetic agrochemicals despite recognized problems for the environment and organisms [10,11,12] because of with lack of safer products and eco-friendly and holistic strategies. As harmful synthetic chemicals are withdrawn from the market, the search for alternatives such as crop rotation, resistant plant varieties, and biological control agents or their derivatives to control nematodes has intensified [4,11,13,14] *Trichoderma* species that have lethal effects against *Meloidogyne* species include *T. harzianum*, *T. koningii*, *T. koningiopsis*, *T. longibrachiatum*, *T. citrinoviride* and *T. viride* [15,16,17] against *M. incognita; T. hamatum,* *T. harzianum*, *T. koningii*, *T. koningiopsis*, and *T. viridae* against *M*. *javanica* [15,18,19]; *T. asperellum,* *T. harzianum*, *T. viride* and *T. viride* on *M. hapla* [9]; and *T. harzianum* against *M. enterolobii* [20].

The main mechanisms of action known for *Trichoderma* species are antibiosis, competition for space and nutrients, mycoparasitism, and induction of defense mechanisms. The antibiosis involves the production and secretion of secondary and primary metabolites that inhibit the growth and development of root-knot nematodes [19,21,22]. Regarding the production and secretion of metabolites, approximately 390 non-volatile metabolites have been identified from *Trichoderma* spp. [2,23], but only a few, such as gliotoxin, gliovirin, heptelidic acid, and viridin identified from *T. virens* [3,24], have been reported to have nematicidal activity. Therefore, a systematic bio-guided screening of *Trichoderma* species is a promising option to find novel nematicidal products.

During our ongoing bioprospecting studies in search of natural agrochemical products in the Yucatán península (Mexico), our group isolated 56 native *Trichoderma* species from soils. The foregoing is in response to the need to develop nematicides based on native species adapted to the areas where they are intended to be applied, with the knowledge of the active metabolites produced by fungal strains. These strains were tested for control of *M. incognita*, and 29 of these strains affected the viability of eggs and second-stage juveniles (J2s) in vitro and acted as plant growth promoters [25,26,27]. Moreover, these active *Trichoderma* strains decreased the severity of nematode damage on tomato and improved yields in the greenhouse [18,25].

In the present study, 13 *Trichoderma* strains from Yucatán were selected due to their activity against phytopathogenic fungi, nematodes, and as plant growth promoters. All selected *Trichoderma* strains were cultured in the liquid media potato dextrose broth. From each fungal strain, the culture filtrate, ethyl acetate fraction, and residual filtrate fraction were obtained and tested against the J2s of *M. incognita* and *M. javanica*. The chemical profiles of the most-active filtrates or fractions were analyzed by liquid chromatography– diode array detector-electrospray-high resolution mass spectrometry (LC-DAD-ESI-HRMS).

## 2. Materials and Methods

### 2.1. Trichoderma Strains

**Figure 1:**
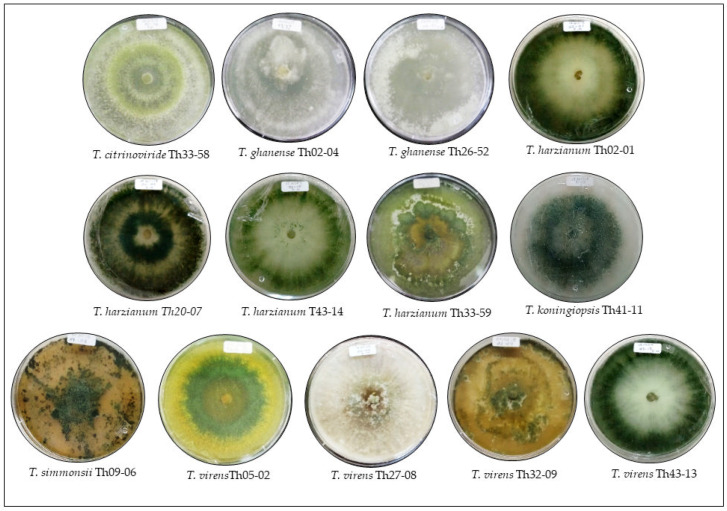
Cultures of the 13 study strains of *Trichoderma* on potato dextrose agar incubated at 25 ± 2 °C, 12 h light/12 h dark for 8 days.

The 13 *Trichoderma* spp. strains, obtained from cultivated and non-cultivated soil in Yucatán state in Mexico [25,26] and supplied by the Phytopathology Laboratory of Tecnológico Nacional de México, Campus Conkal (Table 1), belonged to six *Trichoderma* species: *T*. *citrinoviride* (Th33-58), *T*. *ghanense* (Th02-04, Th26-52), *T*. *harzianum* (Th02-01, Th20-07, Th43-14 and Th33-59), *T*. *koningiopsis* Th41-11, *T*. *simmonsii* Th09-06; and *T. virens* (Th05-02, Th27-08, Th32-09, and Th43-13). The 5.8S-ITS regions of each strain were sequenced previously and are available in the GenBank database [27,28]. The fungal strains were reactivated in Petri dishes with potato dextrose agar (PDA, Dibico, Edo. Mex., MX) and incubated at 25 ± 2 °C, 12/12 h of light/dark for 8 days (Table 1, Figure 1).

#### Liquid Culture of Trichoderma Strains

The strains were grown in potato dextrose broth (PDB), which was made by adding 200 g of potato fragmented in distilled water at the boiling point (1000 mL) for 15 min, then filtered and 20 g of dextrose (Difco, Baltimore, MD, USA) added. A volume of 200 mL of the medium was deposited in Roux bottles and sterilized in an autoclave at 121 °C, 15 lb pressure, for 15 min. For each *Trichoderma* strain, cultured in PDA (8 days), a mycelial disk (7 mm diameter) was added to the medium in each of three Roux bottles. PDB without a *Trichoderma* strain was used as a control (blank). Three replicates of these cultures were incubated at 25 ± 2 °C, 12/12 h light/dark for 31 days. The mycelium was then removed from the culture broth by filtration through a double layer of cheesecloth. Each culture filtrate (CF), designated as 100% concentration, was then diluted with distilled water to 50% and 25% concentrations. The pH of 5 mL of the 100% CF was measured, then stored at 4 ± 2 °C until organic extraction (1–3 days).

### 2.2. Preparation of Fractions from Culture Filtrates

Each CF was liquid–liquid extracted with ethyl acetate three times (2:1, 1:1, 1:1 *v*/*v*), obtaining an ethyl acetate (EA) and residual filtrate (RF) fractions. The EA fraction was dried over sodium sulfate (Merck, New Jersey, USA), and the solvent was vacuum-evaporated at 40 °C in a rotary evaporator (IKA model RV-10, Staufen, Germany). The residual solvent in the RFs was also removed by evaporation, and the residue was designated as 100% concentration. The PDB control was processed the same way. All EAs were stored at 4 °C, and the RF fractions were frozen. 

### 2.3. Nematicidal Bioassay 

#### 2.3.1. Nematode Inoculum 

The population of *M. incognita* was obtained from the Tecnológico Nacional de México/ campus Conkal, Yucatán, México (30 ± 2 °C, 90% relative humidity) and *M. javanica* from the Instituto de Ciencias Agrarias, CSIC in Madrid, Spain (25 ± 1 °C, 70% relative humidity). Both nematodes were maintained on tomato plants (variety Marmanded) growing in pots in a greenhouse. Egg masses were collected from infested tomato roots and incubated for 72 h in sterile distilled water at 25 ± 2 °C for *M. javanica* and 30 ± 2 °C for *M. incognita*. The hatched J2s of *Meloidogyne* were adjusted to a final concentration of 100 J2 nematodes/100 μL distilled water to test CFs and RFs fractions (aqueous samples) and to 100 J2 nematodes/95 μL of distilled water solution to test EA samples [29,30]. 

#### 2.3.2. Sample Preparation and Nematicidal Bioassay

Aqueous samples (100 µL) of a serial dilution (100, 50, or 25%) of either the CF or RF samples of a *Trichoderma* strain or a blank were deposited in wells of 96-well plates with U-bottom (BD Falcon, San Jose, CA, USA). The J2s suspended in distilled water (100 μL) that had been filtered through a 25 µm mesh screen were then transferred into each well. The negative controls consisted of CF or RF blanks, distilled water (DW), and 100 J2s. EA samples (5 µL) dissolved in DMSO:0.6%-Tween 20 (DT) were transferred to each well containing nematode suspension (95 µL), with a final concentration of 1 µg/µL. In this case, the negative control consisted of blank extracts, a mixture of water-DT 95:5 (WDT), and 100 J2s. Four replicates for each treatment were performed. The experimental plates were sealed with parafilm to prevent evaporation and maintained at the same conditions described above for egg masses in the dark [29,31]. 

After 72 h, immobile and rigid J2s that lacked intestinal contents were counted as dead, using a binocular microscope, and expressed as the percentage of juvenile mortality (%M). The nematicidal activity data were corrected using Schneider–Orelli’s formula [32]. A completely randomized design was used, and means were compared using the Scott-Knott test (*p* ≤ 0.05) in the statistical package Infostat Ver. 2018 [33]. 

### 2.4. Liquid Chromatography-Diode Array Detector-Electrospray-High Resolution Mass Spectrometry 

The active EA and RF fractions were freeze-dried (Labconco FreeZone 2.5, model 7670520, Houston, TX, USA) and dissolved to 1% w/v with methanol, then 3 µL was injected onto a C8 column (Zorbax SB, 2.1 × 30 mm) in an Agilent 1200 liquid chromatograph (LC, Santa Clara, CA, USA) coupled to an Agilent diode array detector and a Bruker Maxis HR-QTOF mass detector (HRMS Bruker GmbH, Bremen, Germany). The samples were separated at 40 °C with a flow of 300 μL/min. The mobile phase was a mixture of water–acetonitrile 90:10 *v*/*v* 0.01% trifluoroacetic acid and 1.3 mM ammonium formate (solvent A) and 10:90 *v*/*v* 0.01% trifluoroacetic acid and 1.3 mM ammonium formate (solvent B). The gradient was from 10% to 100% of solvent B in 6 min, maintained in 100% B for 2 min, then returned to 10% B for 2 min [34]. Mass spectra (50 to 2000 *m*/*z*) in the positive mode were acquired, and components detected were compared against the MEDINA Microbial Dereplication Databases (with approximately 900 known bioactive molecules), the Chapman & Hall Dictionary of Natural Products (v25.1, CRC Press, Boca Raton, FL, USA), and a database available in the literature. 

## 3. Results

### 3.1. Nematicidal Activity

**Figure 2:**
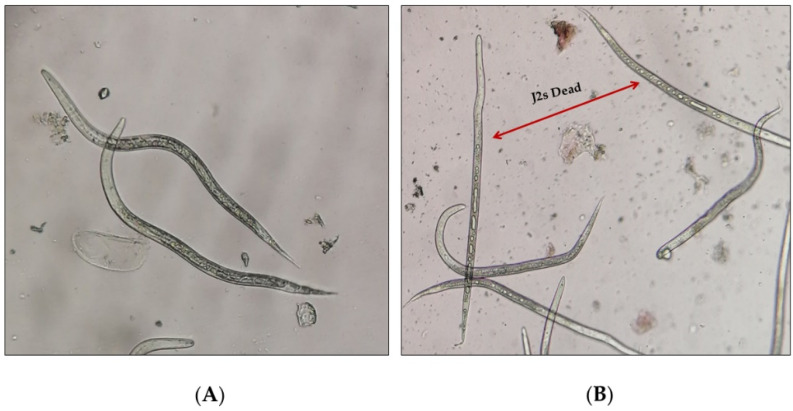
Second-stage juveniles (J2s) of *Meloidogyne incognita* in (**A**) control in distilled water, (**B**) dead and living in culture filtrate of *Trichoderma virens* Th27-08.

The lethality of the CFs of the 13 species of *Trichoderma* against *M. incognita* differed (*p* ≤ 0.05) from that of the negative controls at the 100% concentration (Figure 2, Table 2). All the CFs were mortal to *M. incognita* at 100% concentration, except for that of *T. citrinoviride* (M = 62%) after 72 h. The most mortal CFs at 50% concentration against *M. incognita* after 72 h were from *T. ghanense* Th02-04 (M = 100%), *T. virens* Th27-08 (M = 71%), and *T. harzianum* Th43-14 (M = 66%). The activity of the CFs decreased to M < 50%) at 25% dilution, so they were considered non-nematicidal at this dilution. Against J2s of *M. javanica*, however, only CFs from *T. ghanense* Th02-04, *T. harzianum* Th20-07, and *T. virens* Th27-08 (M values of 85–99%) were effective at 100% concentration after 72 h (Table 2). The nematicidal activity of the CFs at 50% and 25% dilution against *M. javanica* did not differ from the DW. Therefore, the 100% concentration CF from *T. ghanense* Th02-04 was the most effective against both nematodes (M = 99–100%).

After the ethyl acetate extraction of the CFs, the activity of all the RF fractions differed greatly (*p* ≤ 0.05) from that of the negative control. All the residual filtrates generated from the ethyl acetate extraction of the CFs of the 13 strains were mortal against *M. incognita*, and all but those from *T. harzianum* Th20-07 and *T. simmonsii* Th09-06 were mortal against *M. javanica* (Table 2). The highest mortality against J2s of *M. incognita* at 25% dilution of the RFs after 72 h was caused by *T. koningiopsis* Th41-11 (M = 82%) and *T. virens* Th32-09 (M = 83%). Against J2s of *M. javanica*, the highest mortality was achieved with 50% dilutions of the RFs from *T. ghanense* Th26-52 (M = 63%), *T. koningiopsis* Th41-11 (M = 51%) and *T. virens* Th27-08 (M = 94%). After a 72 h exposure to EA fractions, the most active against *M. incognita* were from *T. harzianum* Th43-14 (M = 51%), *T. koningiopsis* Th41-11 (M = 54%), and *T. virens* Th32-09 (M = 100%) at 1 µg/µL; the EA fraction from *T. harzianum* Th43-14 strain was the only EA fraction active (M = 66%) against *M. javanica* at 1 µg/µL (Table 2).

In general, against both nematodes, the CFs and their RF and EA fractions from *T. harzianum* Th43-14 and *T. virens* Th27-08 were the most active. In addition, the RF fraction from CF of *T. koningiopsis* induced J2s mortality of both nematodes at the lowest dilution tested.

### 3.2. Identification of Components in Active Fractions from Trichoderma Strains by LC-DAD-ESI-HRMS

The results obtained from analyses of the liquid chromatograms and ultraviolet and high-resolution mass spectrometry data of the RF fraction from *T. harzianum* Th43-14, *T. ghanense* Th26-52, and *T. virens* Th27-08 and the EA fraction of *T. koningiopsis* are shown in Table 3, Table 4, Table 5 and Table 6 No significant differences were observed between the chromatograms from the active strains *T. ghanense* Th02-04 and the *T. virens* Th32-09 and negative control unfermented culture medium.

The compounds were tentatively identified by taking into account the species of the producing strain, the UV spectrum, and the molecular formula assigned through analysis of the protonated and ammonium adducts of each molecule, helped in some cases by the presence of dimers and dehydration products, and through searches of in the internal MEDINA database of HRMS spectra, dictionary of natural products and other natural product databases.

#### 3.2.1. Metabolites from Residual Filtrate Fraction of *Trichoderma harzianum* Th43-14 

**Figure 3:**
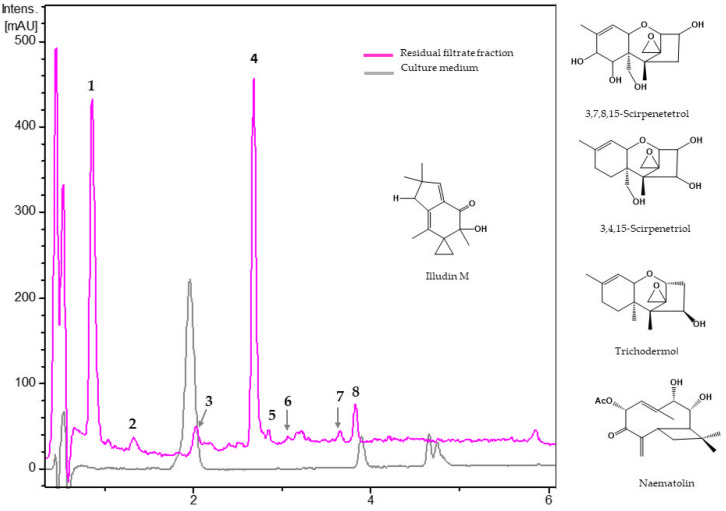
UV_210 nm_ chromatograms of residual filtrate fraction from *Trichoderma harzianum* Th43-14 and unfermented culture medium. **1**: Not identified (C_15_H_24_O_7_), **2**: 3,7,8,15-scirpenetetrol, **3**: illudin M, **4**: not identified (C_14_H_22_O_4_), **5**: 3,4,15-scirpenetriol, **6**: naematolin, **7**: not identified (C_29_H_44_O_10_), **8**: trichodermol.

The chromatogram of the RF fraction from *T. harzianum* Th43-14 showed eight main components (Figure 3, Table 3). Five of these were tentatively identified as sesquiterpenes according to their UV and HRMS data. Component **5** (R*t* = 2.86 min) presented in its HRMS an ammonium adduct (*m*/*z* 300.1807), a protonated molecular ion at *m*/*z* 283.1541, indicative of a molecular formula C_15_H_22_O_5_ (calc. for C_15_H_23_O_5_^+^_,_ 283.1540), which was tentatively assigned as 3,4,15-scirpenetriol. The HRMS of component **2** (R*t* = 1.35 min) exhibited an ammonium adduct (*m*/*z* 316.1758), and a protonated molecular ion at *m*/*z* 299.1492 consistent with the molecular formula C_15_H_22_O_6_ (calc. C_15_H_23_O_6_^+^, 299.1489) and was putatively identified as 3,7,8,15-scirpenetetrol. All compounds tentatively identified (**2**, **5,** and **8**) have the same structural skeleton. 

The HRMS spectrum of the minor component **3** (R*t* = 2.05 min) displayed several protonated fragments (*m*/*z*, 231.1383, 221.1544, 213.1272) and a protonated molecular ion at *m*/*z* 249.1488 in agreement with the molecular formula C_15_H_22_O_6_ (calc. for C_15_H_23_O_6_^+^, 249.1489) and was putatively identified as illudin M. The HRMS spectrum of another minor component (**6**, R*t* = 3.09 min) revealed a protonated molecular ion at *m*/*z* 249.1488 and was given a molecular formula C_15_H_20_O_3_ (calc. for C_15_H_21_O_3_^+^, 249.1485) and tentatively identified as naematolin.

The largest peaks were from two unknown molecules (**1** and **4**), which according to their protonated molecular adducts (*m*/*z* 317.1599 and 255.1591, respectively), ammonium (*m*/*z* 334.1866 and 272.186, respectively), and [2M+ H]^+^ (*m*/*z* 650.3387 and 509.3114, respectively) ions, have molecular formulae of C_15_H_24_O_7_ (R*t* = 0.88 min) and C_14_H_22_O_4_ (R*t* = 2.69 min). In addition, another non-identified minor component (**7**, R*t* = 2.16 min) was assigned a molecular formula of C_29_H_44_O_10_ (R*t* = 3.66 min) based on the analysis of its protonated (*m*/*z* 553.3000) and ammonium adducts (*m*/*z* 570.3270).

**Table 3:** Dereplicated metabolites from residual filtrate fraction of *Trichoderma harzianum* Th43-14.

No.	R*t* (min)	UV (nm)	[M + H]^+^ *m*/*z*		Molecular Formula	Putative Metabolite
			Experimental	Theoretical		
1	0.88	200, 215	317.1599	317.1595	C_15_H_24_O_7_	unknown
2	1.35	205, 210	299.1492	299.1489	C_15_H_22_O_6_	3,7,8,15-Scirpenetetrol
3	2.05	200, 220	249.1488	249.1485	C_15_H_20_O_3_	Illudin M
4	2.69	200, 220	255.1591	255.1591	C_14_H_22_O_4_	unknown
5	2.86	200, 220	283.1541	283.1540	C_15_H_22_O_5_	3,4,15-Scirpenetriol
6	3.09	200, 218, 270	309.1692	309.1697	C_17_H_24_O_5_	Naematolin
7	3.66	200, 220	553.3000	553.3007	C_29_H_44_O_10_	unknown
8	3.84	200, 220	251.1644	251.1642	C_15_H_22_O_3_	Trichodermol

No.: Component number; Rt: Retention time; UV: Ultraviolet.

#### 3.2.2. Metabolites from Residual Filtrate Fraction of *Trichoderma ghanense* Th26-52

**Figure 4:**
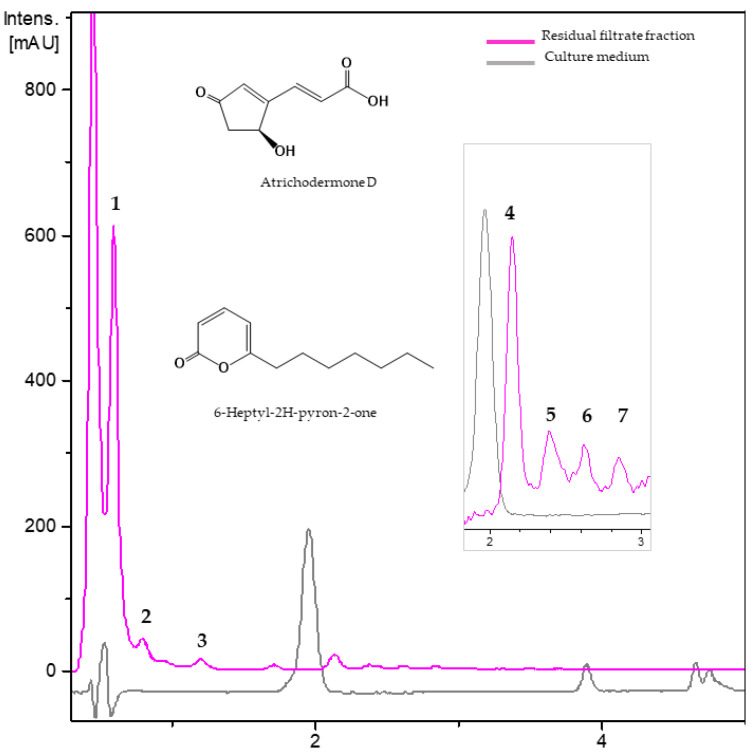
UV_210 nm_ chromatograms of residual filtrate fraction from *Trichoderma* *ghanense* Th26-52 and the unfermented culture medium. **1**: Not identified (C_11_H_16_O_7_), **2**: not identified C_8_H_9_NO_3_, **3**: not identified C_8_H_10_O_3_, **4**: atrichodermone D, **5**: Not identified (C_11_H_12_O_5_), **6**: not identified (C_14_H_18_O_3_). **7**: 6-heptyl-2H-pyron-2-one.

Seven components were detected in the chromatogram of the residual fraction of *T. ghanense* Th26-52 (Figure 4, Table 4). The most abundant component, **1** (R*t* = 0.63 min) displayed in its HRMS spectrum an ammonium adduct (*m*/*z* 278.1233) and a protonated molecular ion at *m*/*z* 261.0966, indicative of a molecular formula of C_11_H_16_O_7_ (calc. C_11_H_16_O_7_^+^, 261. 0973); this polar metabolite could not be identified. The HRMS of component **4** (R*t* = 2.16 min) with a protonated molecular ion at *m*/*z* 169.0493 has a molecular formula C_8_H_8_O_4_ (calc. C_8_H_9_O_4_^+^, 169.0500) and was putatively identified as atrichodermone D. The analysis of the HRMS data of the minor component **7** (R*t* = 2.89 min) displayed an ammonium adduct (*m*/*z* 212.1640), and a molecular protonated ion at *m*/*z* 195.1379 supporting the molecular formula C_12_H_18_O_2_ (calc. C_12_H_19_O_2_^+^, 195.1384) and tentatively assignment as the unsaturated lactone 6-heptyl-2H-pyron-2one. The other four unknown components included small metabolites with the molecular formulae of C_8_H_9_NO_3_ (R*t* = 0.86 min), C_8_H_10_O_3_ (R*t* = 1.22 min), C_11_H_12_O_5_ R*t* = 2.40 min), and C_14_H_18_O_3_ (R*t* = 2.64 min) according to their UV and HRMS data (Table 4).

**Table 4:** Dereplication metabolites from ethyl acetate fraction of *Trichoderma ghanense* Th26-52.

No.	R*t* (min)	UV (nm)	[M + H]^+^ *m*/*z*		Molecular Formula	Putative Metabolite
			Experimental	Theoretical		
1	0.63	200, 218	261.0966	261.0969	C_11_H_16_O_7_	unknown
2	0.86	218, 230, 300	168.0656	168.0655	C_8_H_9_NO_3_	unknown
3	1.22	205, 290	155.0700	155.0703	C_8_H_10_O_3_	Unknown
4	2.16	200, 220, 310	169.0493	169.0495	C_8_H_8_O_4_	atrichodermone D
5	2.40	200, 210, 240	225.0756	225.0758	C_11_H_12_O_5_	Unknown
6	2.64	200, 230, 270	235.1328	235.1329	C_14_H_18_O_3_	Unknown
7	2.89	200, 220, 280	195.1379	195.1380	C_12_H_18_O_2_	6-heptyl-2H-pyron-2-one

No.: Component number; Rt: Retention time; UV: Ultraviolet.

#### 3.2.3. Metabolites from Ethyl Acetate Fraction of *Trichoderma koningiopsis* Th41-11

**Figure 5:**
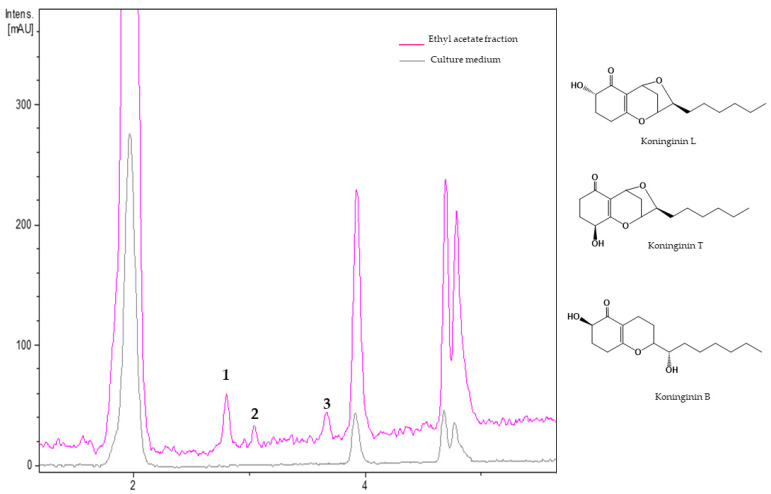
UV_210 nm_ chromatogram of residual filtrate fraction from *Trichoderma koningiopsis* Th41-11 and the unfermented culture medium. **1**: Koninginin T, **2**: koninginin L, **3**: koninginin B.

The chromatogram of the ethyl acetate extract from *T. koningiopsis* Th41-11 (Figure 5) showed three components, which tentatively were assigned as koninginin isomers. The HRMS of component **1** (R*t* = 2.81 min) and **2** (R*t* = 3.04 min) exhibited similar dehydration adducts (*m*/*z* 263.1642 245.1535) and protonated adducts (*m*/*z* 281.1750 and 281.1749, respectively), indicative of a molecular formula of C_16_H_24_O_4_ (calc. for C_16_H_25_O_4_+, 281.1752) for both molecules. Component **3** (R*t* = 3.68 min) in its HRMS data showed dehydrated species (*m*/*z* 265.1796, 247.1668, 237.1847), a dimer adduct ([2M+H]^+^, *m*/*z* 565.3759), and a protonated molecular ion at *m*/*z* 283.1906 in accordance with a molecular formula of C_16_H_26_O_4_ (calc. for C_16_H_27_O_4_^+^, 283.1908). An extensive search of the literature based on spectral data and previous reports led to their tentatively identification as koninginin L (**1**), T (**2**), and B (**3**) (Table 5).

**Table 5:** Dereplicated metabolites from ethyl acetate fraction of *Trichoderma koningiopsis* Th41-11.

No.	R*t* (min)	UV (nm)	[M + H]^+^ *m*/*z*		Molecular Formula	Putative Metabolite
			Experimental	Theoretical		
1	2.81	200, 255	281.1750	281.1747	C_16_H_24_O_4_	Koninginin L
2	3.04	200, 255	281.1749	281.1747	C_16_H_24_O_4_	Koninginin T
3	3.68	200, 260	283.1906	283.1904	C_16_H_26_O_4_	Koninginin B

No.: Component number; Rt: Retention time; UV: Ultraviolet.

#### 3.2.4. Metabolites from Residual Filtrate Fraction of *Trichoderma virens* Th27-08

**Figure 6:**
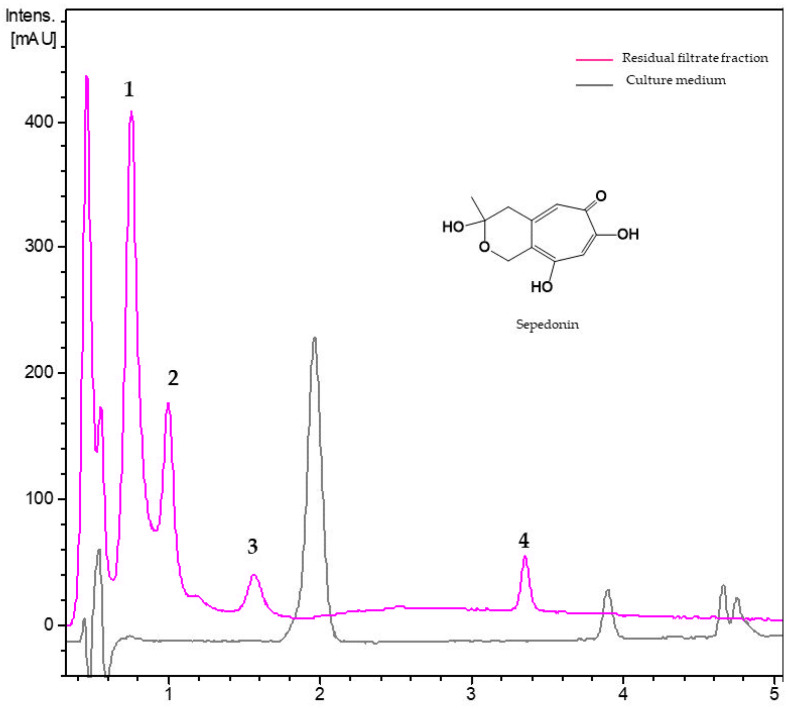
UV_210 nm_ chromatogram of residual filtrate fraction from *Trichoderma virens* Th27-08 and the unfermented culture medium. **1**: Not identified (C_11_H_10_O_4_), **2**: sepedonin, **3**: not identified (C_11_H_10_O_4_), **4**: not identified (C_16_H_20_O_4_).

The chromatogram of RF fraction of *T. virens* Th27-08 displayed four components not present in the blank sample (Figure 6, Table 6). Component **3,** eluting at R*t* of 1.00 min exhibited an ammonium adduct (*m*/*z* 242.1020), dehydration fragments (*m*/*z* 207.0652, 191.1424), and a protonated ion at *m*/*z* 225.0757 indicative of a molecular formula of C_11_H_12_O_5_ (calc. for C_11_H_13_O_5_^+^, 225.0762). Based on HRMS and UV data, compound **1** was tentatively identified as sepedonin. The major (**1**) and two minor components (**2** and **4**) were not identified, and molecular formulae were assigned as C_11_H_10_O_4_ (R*t* = 0.78 and 1.57 min) and C_16_H_20_O_4_ (R*t* = 3.35) based on their protonated HRMS ion [M+H]^+^ and additionally supported by its ammonium and dimer adducts.

**Table 6:** Dereplicated metabolites from residual fractions of *Trichoderma virens* Th27-08.

No.	R*t* (min)	UV (nm)	[M + H]^+^ *m*/*z*		Molecular Formula	Putative Metabolite
			Experimental	Theoretical		
1	0.78	205, 240, 290	207.0653	207.0657	C_11_H_10_O_4_	unknown
2	1.00	210, 225, 295,	225.0757	225.0762	C_11_H_12_O_5_	sepedonin
3	1.57	205, 240, 290	207.0652	207.0657	C_11_H_10_O_4_	unknown
4	3.35	215, 255, 300	277.1435	277.1438	C_16_H_20_O_4_	unknown

No.: Component number; Rt: Retention time; UV: Ultraviolet.

## 4. Discussion

The results of this study complement our previous discoveries on native *Trichoderma* strains with nematicidal properties against two *Meloidogyne* species as part of our continuing work to find and develop safer biocontrol agents. Herein, we demonstrated that 92% of the screened tropical *Trichoderma* strains (all 13 except T. *simmonsii*) are mortal to J2s of *Meloidogyne javanica* and confirmed that the CFs and the EA or RF fractions of all strains were highly mortal to *M. incognita*. *T. koningiopsis* UFSMQ40 [12], *T. harzianum* Th6, JX1614550 [18,19,35], and *Trichoderma* sp. EF1671 [5] have been shown to have nematicidal effects against J2s of *M. javanica*, but our report is the first to demonstrate the nematicidal activity of *T. citrinoviride, T. ghanense,* and *T. virens* against *M. javanica*. 

The mortality data revealed that *M. javanica* was less sensitive than *M. incognita* to the CFs and EA fraction from *Trichoderma* strains. This differential sensitivity behavior of both *Meloidogyne* species against extracts, compounds, or fungal strains has been previously reported. For example, fungus *Arthobortys* sp. MVD18 caused less mortality against *M. javanica* (92.9%) than against *M. incognita* (99.0%) after a 48 h exposure. However, two *Trichoderma* sp. strains (KAV2 and KAV3) were more active against *M. javanica* than *M. incognita* [36]. Acetic acid and hexanoic acid yielded an EC_50_ of 162.4 and 339.3 µg/mL, respectively, against *M. javanica* but EC_50_ of 38.3 and 41.1 µg/mL against *M. incognita*, respectively, after 1 day [37,38]. Sensitivity differences between different species and even different populations of the same species have been attributed to the habitat and environmental conditions to which the organisms are exposed. Other saprophytic fungi have been reported to have low nematicidal effects against J2s of *M. javanica*; for example, a CF of *Arthrobotrys oligospora*, *A. conoides,* and *Hypocrea lixii* (sexual state of *T. harzianum*) at 100% concentration [16,39] caused from 16.14 to 64.5% mortality. An EA extract from *Trichoderma* sp. EFI 671, however, was not active [5].

The present study is also the first nematicidal bio-guided fractionation of the CFs from *Trichoderma* species and screening the organic and residual aqueous fractions against J2s. After the fractionation, 69% of the RF fractions from the non-nematicidal CFs had a higher mortal effect on *M. javanica*. This effect could be attributed to antagonistic action between metabolites and the concentration of the metabolites in the RFs of the strains. By contrast, the nematicidal effect of the CF from *T. harzianum* strain Th20-07 was not confirmed, suggesting that enzymes were mainly involved in the nematicidal activity of strain Th20-07 and were subsequently denatured by the solvent during the fractionation. The antibiosis produced by chitinase and protease action from *Trichoderma* species has been widely described. For example, an enzymatic filtrate obtained from *T. koningiopsis* UFSMQ40 is mortal to J2s of *M. incognita* (90.4% mortality) and *M. javanica* (63.2%) after 24 h of exposure [15]. Chitinase (51.42 U/mL) and protease (4.27 U/mL) from *T. harzianum* ITCC 6888 caused high mortality (93%) against *M. incognita* [40].

On the other hand, in our study, the highest lethal activity against both *Meloidogyne* species was found for *T*. *koningiopsis* Th41-11, *T. harzianum* Th43-14, *T. virens* Th27-08, and the two *T. ghanense* strains tested. The most investigated of these species has been *T. harzianum* for its properties as a biological control agent and its metabolites [2,4,14]. Other in vitro studies of CFs from *T. harzianum* strains grown in PDB have shown a significant mortal effect on the J2s of *M. incognita*. For example, CFs from *T. harzianum* strains ThU, JX1614550 and Th.6 caused a mortality of 33, 64.5 and 75%, respectively, at 100% concentrations after 72 h [19,41,42].

The residual fraction of *T. harzianum* Th43-14 revealed a mixture of illudin, naematolin, 4β-scirpenol (syn. trichodermol and roridin C), 3,4,15-scirpenetriol, and 3,7,8,15-scirpenetetrol were tentatively identified based on UV and MS data. Scirpenes have a trichothecene skeleton with a characteristic 9-double-bond and C12-C13 epoxide in their structure and are potent inhibitors of protein synthesis [43,44]. Semisynthetic derivatives of trichodermol have potent cytotoxic and antifungal activity against the fungus *Ceratocystiopsis crassivaginata* [45,46]. This metabolite has been isolated from several *Trichoderma* species, and 3,4,15-scirpenetriol has been isolated from *Fusarium equiseti*, *F. roseum*, and *F. sporotrichiella*, whereas 3,7,8,15-scirpenetetrol has only been isolated from *F. graminearum* [47]. Illudin M, isolated from several *Omphalatus* species and from *Granulobasidium vellereum*, has no activity against *M. incognita* or *Caenorhabditis elegans* at 100 µg/mL, but it has high antitumor and antimicrobial activities [48,49,50]. Naematolin was isolated from *Hypholoma fasciculare* (syn. *Naematoloma fasciculare*), a poisonous basidiomycete [51]. This caryophyllenediol derivative has antitumor, antiviral, and weak antibacterial properties [52,53] and reduced 97% of bloodstream forms of *Trypanosoma cruzi* at 250 µL/mL [54]. 

The two studied strains of *T. ghanense* (Th02-04 and Th26-52) were active on both *Meloidogyne* species. The data obtained from the UV and ESIMS spectra of its RF fraction allowed us to tentatively identify atrichodermone D and 6-heptyl-2H-pyron-2-one in the RF fraction of *T. ghanense* Th26-52. To date, metabolites detected from *T. ghanense* include the phenolics catechin, ferulic acid, and cinnamic acid from the CF of two strains from different areas in India [55]. Atrichodermone D is a cyclopentenone previously reported only from *T. atroviride* and has no known biological activity [56]. 6-Heptyl-2H-pyran-2-one is an alkyl pyrone isolated from *T. koningii* strain IMI-308475 and from *T. asperellum* and *T. viride*, but no nematicidal activity was reported [57,58]. Related lactones from *Trichoderma* species include 6-(1-heptenyl)-2-pyran-2-one, 6-(3-hydroxypent-1-en-1-yl)-2H-pyran-2-one and 6-pentyl-2H-pyran-2-one. 6-(3-Hydroxypent-1-en-1-yl)-2H-pyran-2-one was isolated from *T. koningiopsis* QA3 and has strong antibacterial activity against *Micrococcus luteus* [59]. 6-Pentyl-2H-pyran-2-one is a recognized plant growth promoter and produced by several *Trichoderma* species [60]. 

From the results of the present study, *T*. *koningiopsis* Th41-11 is another promising strain that produces nematicidal metabolites. The LC chromatogram of its EA fraction revealed small amounts of koninginins B, L, and T, tentatively identified based on UV, ESIHRMS data analyses, and comparisons with the literature. Koninginin B is a bicyclic polyketide that has also been reported from *T. koningii* [61], *T. neokoningin* [62], and *T. applanatum* [63]. Koninginin L and T are tricyclic polyketides with an oxygen bridge (C7 and C9) at C2 and an alcohol group at C4. Koninginins B, L, and T from *T. koningiopsis* QA3 and YIM PH30002 were recently reported to be weakly antibacterial [64,65,66].

Among the four *T. virens* strains studies, only Th27-08 had activity against both nematodes, with the RF fraction achieving the highest mortality. In the LC chromatogram of the RF fraction, we detected sepedonin, a tropolone first isolated from *Sepedonium chrysospermum* Fries (teleomorph *Hyphomyces chrysospermus* Tul.) and later from *S. ampullosporum*, *S. chalcipori*, *S. microspermum*, and *S. chrysospermum* and having antimicrobial activity against several bacterial and fungal pathogens [67]. The artifact anhydrosepedonin (C_11_H_10_O_4_) is produced during the isolation process due to the instability of sepedonin [68,69]. We additionally detected three unknown metabolites in the RF fraction of *T. virens* Th27-08. Other metabolites previously reported from *T. virens* include cathequin, caffeic acid, ferulic acid, and 33 other non-volatile metabolites [2,55,70,71]. Our report of sepedonin is thus a new contribution to the chemical composition of this species.

Except for illudin M, the metabolites reported from our native *Trichoderma* species were not previously tested on nematodes [48]. The nematicidal efficacy of the metabolites tentatively identified from *Trichoderma* species herein studied are likely due to the alcohol or carboxylic acid groups in their structure. Ntalli et al. [38] reported that acetic acid and hexanoic acid, furfurol (syn. furfuryl alcohol), and furfural paralyzed J2s an EC_50/1 h_ of 1–100 µg/mL or less after 24 h, and the alcohols and aldehyde were more effective than the organic acids. They also demonstrated that acetic acid damages the cuticle and, the nuclei of pseudocoel cells and vacuolizes the cytoplasm of *M. incognita,* while (*E)*-2-decenal, and undecanone induced malformation of somatic muscles of the nematodes [72].

In general, few metabolites with nematicidal properties have been isolated from *Trichoderma* species; some of these are acetic acid, gliotoxin, trichorzianine, viridin [3], trichodermin [73], and cyclonerodiol [65,74]. Therefore, more studies should focus on bioassay-guided isolation and characterization of metabolites responsible for nematicidal activity in the fractions from *Trichoderma* strains. In addition, studies to optimize the production of the extracts with the most promising active compounds, evaluate their efficacy in greenhouses, and assess their toxicity on plants and beneficial organisms must be carried out before the compounds can be evaluated in the field and further developed as safe bionematicide products.

## 5. Conclusions

The present contribution enriches our knowledge of the nematicidal potential of 13 tropical *Trichoderma* species isolated from the soils of Yucatán state. The most effective species against *M. incognita* and *M. javanica* were *T. ghanense* strains Th02-04 and Th26-52, *T. harzianum* Th43-14, *T. koningiopsis* Th41-11, and *T. virens* Th27-08. The LC-DAD-ESIMS chemical profiles of the residual filtrate fractions and ethyl acetate fractions of culture filtrate from *Trichoderma* spp. revealed the presence of novel metabolites for the genus and others with molecular formulas not found in natural products databases. These results highlight the ability of *Trichoderma* strains to produce bioactive secondary metabolites that could be developed to manage *M. incognita* and *M. javanica*. However, more studies are needed to determine activity and potential phytotoxicity in plant applications and doses for effective, efficient biocontrol effect.

## Figures and Tables

**Table 1:** *Trichoderma* strains selected and their biological activity.

Trichoderma Species	Key	GenBank Number	Activity	Place of Collection
*T. citrinoviride* Bissett 1984	Th33-58	MF078653	A/B	Ticul
*T. ghanense* Yoshim. Doi, Y. Abe & Sugiy 1987	Th02-04	MF078652	A/B	Tizimín
	Th26-52	MF078651	A/B	Tahdziú
*T. harzianum* Rifai 1969	Th02-01	MF952887	A/B	Tizimín
	Th20-07	MF078650	A	Tzucacab
	Th43-14	MF078649	A	San Felipe
	Th33-59	MF078648	A/B	Ticul
*T. koningiopsis* Samuels, Carm. Suárez & H.C. Evans 2006	Th41-11	MF952888	A/B	Sanahcat
*T. simmonsii* P. Chaverri, F.B. Rocha, Samuels, Degenkolb & Jaklitsch 2015	Th09-06	MF078647	A/B	Dzidzantun
*T. virens* (J.H. Mill., Giddens & A.A. Foster) Arx 1987	Th05-02	MF952889	A/B	Dzilam González
	Th27-08	MF078646	A	Chacsinkin
	Th32-09	MF078645	A	Oxkutzcab
	Th43-13	MF078644	A	San Felipe

A: *Meloidogyne incognita* antagonist; B: plant growth promoter.

**Table 2:** Mortality of second-stage juveniles of *Meloidogyne* spp. after 72 h exposure to culture filtrate (CF), ethyl acetate (EA), or residual filtrate (RF) fractions from tropical *Trichoderma* strains.

*Trichoderma* Species	Strains	Mean Percentage Mortality									
		*Meloidogyne incognita*						*Meloidogyne javanica*			
		CF		EA	RF			CF	EA	RF	
		100%	50%	1 µg/µL	100%	50%	25%	100%	1 µg/µL	100%	50%
*T. citrinoviride*	Th33-58	62 b	21 c	30 c	100 a	66 c	13 c	15 d	8 b	100 a	1 h
*T. ghanense*	Th02-04	100 a	100 a	20 d	100 a	63 c	24 c	99 a	0 c	100 a	16 e
	Th26-52	100 a	23 c	22 d	100 a	100 a	20 c	3 f	11 b	100 a	63 b
*T. harzianum*	Th02-01	100 a	42 c	0 e	100 a	23 d	15 c	4 f	0 c	98 a	34 d
	Th20-07	100 a	6 d	0 e	98 a	66 c	13 c	97 a	0 c	7 e	0 h
	Th43-14	100 a	66 b	51 b	100 a	100 a	57 b	45 c	66 a	93 b	4 g
	Th33-59	100 a	21 c	30 c	100 a	72 b	22 c	9 e	9 b	10 a	0 h
*T. koningiopsis*	Th41-11	100 a	38 c	54 b	100 a	100 a	82 a	7 e	1 c	93 b	51 c
*T. simmonsii*	Th09-06	100 a	6 d	37 c	100 a	96 a	53 b	6 e	0 c	14 d	0 h
*T. virens*	Th05-02	100 a	22 c	18 d	100 a	19 d	18 c	8 e	0 c	100 a	10 f
	Th27-08	100 a	71 b	0 e	100 a	100 a	64 b	85 b	9 b	100 a	94 a
	Th32-09	100 a	20 c	100 a	100 a	100 a	83 a	7 e	3 c	86 c	5 g
	Th43-13	100 a	23 c	31 c	100 a	86 b	49 c	7 e	8 b	95 b	0 h
Control	PDB	0 c	0 d	0 e	0 b	0 d	0 c	0 f	0 c	0 f	0 h
	DW/WDT *	0 c	0 d	0 e*	0 b	0 d	0 c	0 f	0 c*	0 f	0 h
	Blank	0 c	0 d	0 e	0 b	0 d	0 c	0 f	0 c	0 f	0 h
	SD	0.2	125	5.7	0.5	15.2	12.9	2.4	1.4	2.6	2.4

PDB: potato dextrose broth, DW: distilled water, WDT *: water-DMSO–0.6% Tween 20 (95:5); Blank: unfermented medium. SD: standard deviation, calculated from the analysis of variance for treatments (CFs, EA, RFs from each strain). Values with different letters within a column differed significantly in Scott–Knott test (*p* ≤ 0.05).

## Data Availability

Not applicable.
